# Chronic Thoracic Aortic Aneurysm Presenting 29 Years following Trauma

**DOI:** 10.1155/2015/470917

**Published:** 2015-08-13

**Authors:** Sarah Miller, Prashant Kumar, Rene Van den Bosch, Adib Khanafer

**Affiliations:** ^1^General Surgery, Timaru Hospital, South Canterbury 7910, New Zealand; ^2^Emergency Medicine, Timaru Hospital, South Canterbury 7910, New Zealand; ^3^Timaru Hospital, South Canterbury 7910, New Zealand; ^4^Christchurch Hospital, Riccarton Avenue, Christchurch 8053, New Zealand

## Abstract

Blunt, nonpenetrating injuries of the thoracic aorta are uncommon and associated with a high mortality rate within the first hour. Aortic injury is missed in 1-2% of patients that survive to hospital, and a chronic thoracic aortic aneurysm may subsequently form. We present a case in which a chronic thoracic aortic aneurysm was diagnosed 29 years following a significant motor vehicle accident. We discuss the epidemiology, presentation, and management of this uncommon consequence of blunt, nonpenetrating aortic injury. Our case illustrates an important clinical lesson; a past medical history of trauma should not be overlooked at any patient assessment.

## 1. Introduction

The formation of a chronic thoracic aortic aneurysm is an uncommon consequence of blunt, nonpenetrating injury to the thoracic aorta. Mortality from blunt aortic injury is high and only 10–20% of patients survive to hospital. Of these, aortic injury, most frequently caused by rapid deceleration trauma, goes unrecognised in 1-2% and a chronic false aneurysm may subsequently form [1, 2]. Diagnosis of these aneurysms may only occur years later, either due to incidental findings, late rupture, or the development of nonspecific symptoms [2, 3]. We report a case of a 47-year-old man who underwent thoracic endovascular aneurysm repair for a chronic thoracic aortic aneurysm diagnosed 29 years after a significant motor vehicle accident.

## 2. Case Presentation

A 47-year-old white Australian man presented to the emergency department after waking with chest tightness and shortness of breath. He denied symptoms of fever, cough, or night sweats. Initial documentation revealed no significant past medical history. At the age of 18, however, he had been the driver in a single car motor vehicle accident. His car had hit a pole at speed and the patient had sustained bilateral pneumothoraces, liver lacerations, and splenic rupture requiring splenectomy. At that time there was no known aortic injury. He had been wearing a seatbelt. The patient had had no hospital admissions since the time of his accident and was otherwise fit and well. He took no regular medications and was a nonsmoker with no history of illicit drug use. He had no risk factors for cardiac disease or pulmonary embolism and no family history of aneurysmal or cardiac disease. On examination, his temperature was 36.5°C, heart rate 76 bpm, blood pressure 139/64, and saturation 98% on air. Chest and abdominal examination were unremarkable.

**Figure 1:**
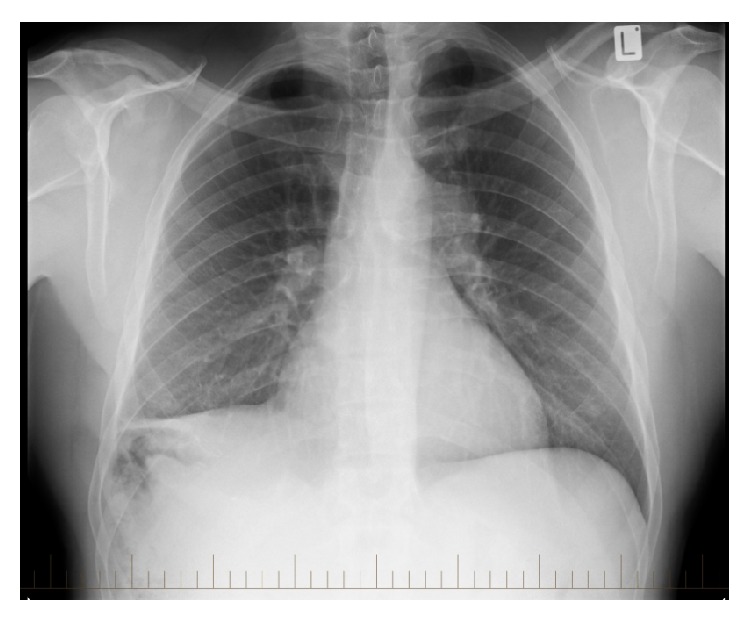
Chest X-ray: lung fields appear clear and there is no evidence of cardiomegaly. A smooth enlargement of the left hilum, initially felt to represent an enlarged pulmonary artery, can be seen.

**Figure 2:**
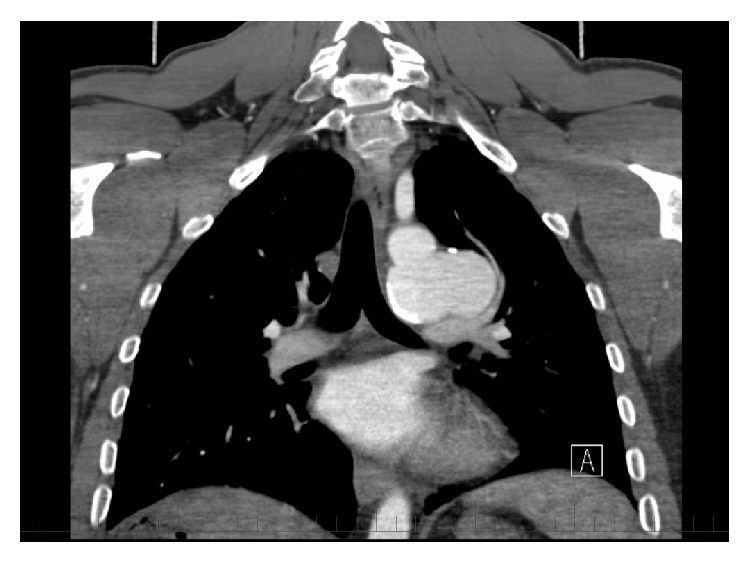
Coronal computed tomography imaging. A 39 mm × 54 mm × 32 mm saccular aneurysm can be seen arising from the aortic isthmus.

**Figure 3:**
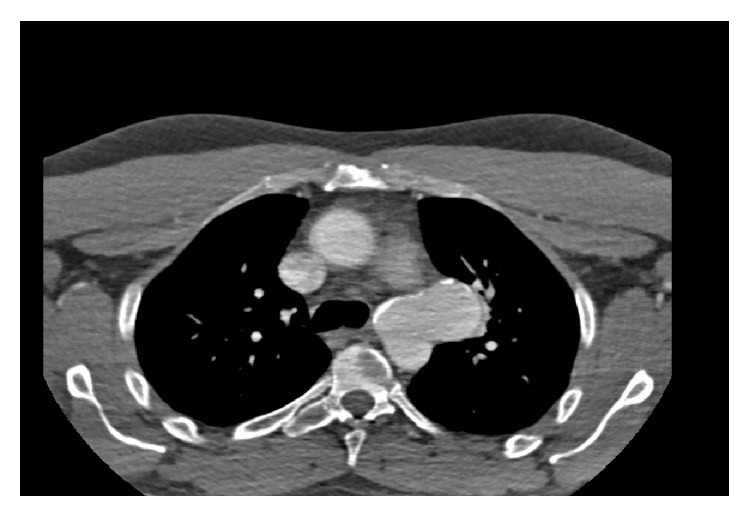
Transverse computed tomography imaging. Dense calcifications can be seen within the aneurysm wall.

**Figure 4:**
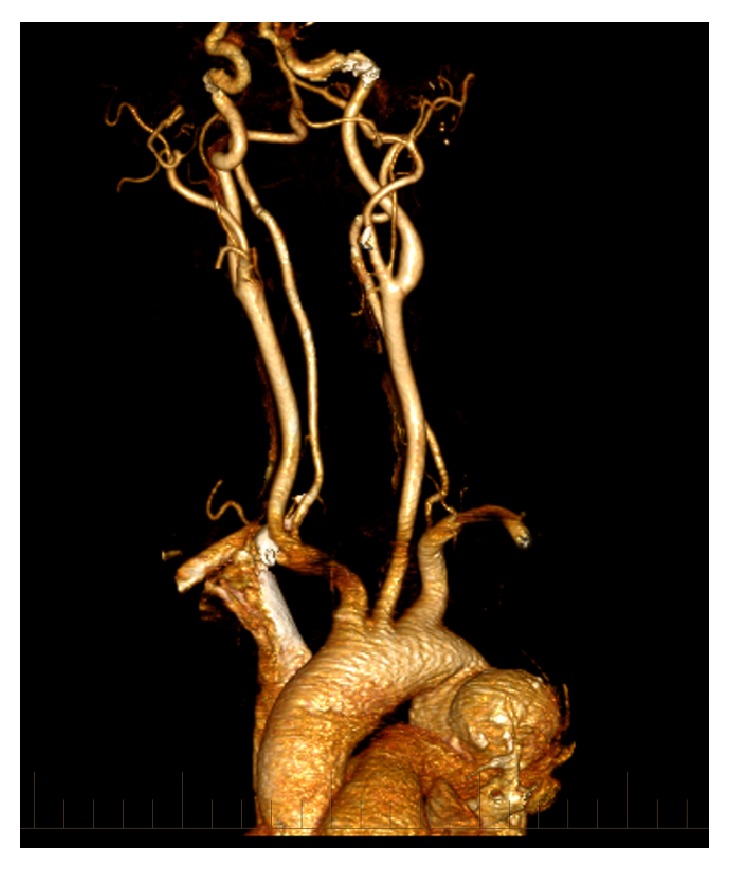
3D reconstruction demonstrating the saccular aneurysm arising from the aortic isthmus.

Electrocardiogram (ECG) demonstrated normal sinus rhythm with no evidence of ischaemia. Routine blood tests including hsTroponin T were within normal range. Chest X-ray (CXR) revealed clear lung fields with a normal cardiothoracic ratio (Figure 1). There was a smooth enlargement of the left hilum on CXR, most likely due to an enlarged main pulmonary artery; further evaluation was advised. Subsequent computed tomography (CT) imaging of the chest revealed however a 5.4 cm saccular thoracic aneurysm originating from the aortic arch near to the left subclavian artery origin (Figures 2, 3, and 4). The appearance of peripheral calcifications suggested that the aneurysm was long-standing and therefore an unlikely cause of the patient's acute symptoms which settled in the emergency department with no specific management. There was no perianeurysmal fluid collection and no evidence of rupture. CT angiogram of the thoracic aorta confirmed the findings and indicated that the proximal margin of the aneurysm lay adjacent to the posterior origin of the left subclavian artery and was 19 mm downstream from the posterior margin of the left common carotid artery. Imaging of the circle of Willis and carotids demonstrated an intact vertebrobasilar confluence.

Differential diagnoses of the cause of the patient's aneurysm required consideration. His past history of trauma was the most likely aetiology. Despite being mildly hypertensive at presentation, there were no subsequent concerns regarding the patient's blood pressure. He was not elderly and had no history of systemic inflammatory disease. Examination was not consistent with Marfan syndrome. The patient had no history of a chronic infectious state such as syphilis, and inflammatory markers were normal. Although CT appearances demonstrated peripheral calcification, this is not an unusual finding in chronic thoracic aneurysms and the patient had no documented history suggestive of atherosclerosis. Furthermore, the aneurysm's saccular shape and location were typical of those seen in chronic traumatic thoracic aortic aneurysms.

Six weeks following his initial presentation, the patient underwent elective thoracic endovascular aneurysm repair (TEVAR) with debranching of the left aortic arch by right carotid to left carotid to left subclavian bypass. There were no immediate postoperative complications. At twelve weeks after procedure the patient returned to work as an emergency department nurse and at nine months following his initial presentation he remains well.

## 3. Discussion

Blunt, nonpenetrating injuries of the thoracic aorta are uncommon but associated with a mortality rate of approximately 80% within the first hour [3, 4]. Deceleration injury as a consequence of road traffic accident is the most common cause of blunt traumatic aortic injury. Other causes include direct blows or falls from significant height [1, 5]. Of those patients that reach hospital, there is over 85% mortality within ten weeks if no treatment is initiated [6]. In 1-2% however, where traumatic aortic injury initially goes unrecognised, a chronic traumatic aortic aneurysm may form [3, 4]. We anticipate that the natural history of chronic traumatic aortic aneurysms may change given the current availability and use of CT imaging in early trauma care; as the number of unrecognised aortic injuries decreases, the diagnosis of longstanding chronic traumatic aortic aneurysm is likely to become increasingly rare.

Rapid deceleration and the application of shearing forces mean that thoracic aortic injuries typically arise from the isthmus, the area distal to the left subclavian artery and proximal to the third intercostal artery [1, 2, 5]. The vulnerability of this anatomical area is likely to be related to the tethering of the descending aorta by the ligamentum arteriosum; however several other mechanisms including the “water-hammer” theory and “osseous pinch” theory have been postulated [3].

Presenting symptoms of chronic traumatic aortic aneurysms may be nonspecific and include haemoptysis, thoracic pain, hoarseness, and back pain. Frequently they are an incidental finding, although they may present with late rupture [2, 3]. A high index of suspicion is usually required for diagnosis and routine and detailed questioning of past trauma history is therefore essential at all patient encounters and should not be overlooked. Careful history taking enables radiological suspicions to be put in the context of a clinical history.

In acute blunt aortic trauma, management of aortic injuries depends on the haemodynamic stability of the patient and the injury severity grading [7]. Haemodynamically unstable patients should be taken for urgent surgery and if the source of haemodynamic instability is found to be aortic trauma, this should be repaired immediately. In haemodynamically stable patients, Type 1 injuries (intimal tears) can be managed nonoperatively with aggressive heart rate and blood pressure control and serial imaging [7]. Type II (intramural haematoma), Type III (pseudoaneurysm), and Type VI (rupture) injuries should be repaired [7]. The Society for Vascular Surgery currently recommends endovascular repair of traumatic thoracic aortic injury in a stable patient within 24 hours and at the latest prior to hospital discharge [7].

Few guidelines focus specifically on the management of chronic thoracic aneurysms. In the majority of cases management is therefore similar to that of thoracic aneurysms of other causes, whereby surgical intervention is based upon patient symptoms and documented radiological enlargement [3, 4]. Katsumata et al. argue however that the maximum diameter and saccular shape of a chronic aneurysm are not strong predictors of rupture as densely calcified traumatic aneurysms may offer significant protection against rupture due to increased wall strength [3]. As such, intervention may not be required although serial follow-up imaging would be recommended [3]. In our case, a decision was made to proceed with surgical repair due to our patient's relatively young age and anxiety about his diagnosis.

As with the management of acute aortic trauma, surgical options include conventional open surgery or thoracic endovascular aneurysm repair (TEVAR). Conventional surgical repair involves a high posterolateral thoracotomy with or without cardiopulmonary bypass [7]. Complications include spinal cord injury, bleeding, and stroke [1, 6, 7]. The less invasive approach of TEVAR involves the implantation of a stent graft across the aneurysm under imaging guidance [5, 7]. Mortality is significantly lower when compared to open repair, with a lower risk of spinal cord ischaemia, stroke, and renal failure [5, 7]. The risk of endoleak, stent collapse, and importance of anatomic suitability for graft placement are significant challenges however, and debranching procedures enabling a sufficient proximal landing zone, particularly when performed across the midline as in our patient, are not without risk. Optimal follow-up strategies are also not established and may constitute significant expense [7]. Data recently published by Szostek et al. that focuses on the endovascular management of chronic traumatic thoracic aneurysms in particular indicates that endovascular repair of these aneurysms has significant advantages, although it is not without complications and long-term outcomes remain unknown [8]. We propose that further research involving patients such as ours is required in order to establish up-to-date guidelines for the specific management of chronic thoracic aortic aneurysms.

In conclusion, this case illustrates an uncommon but important consequence of blunt, nonpenetrating injury to the thoracic aorta. It highlights an important clinical lesson for clinicians at all levels; a background of significant trauma is an important part of a patient's past medical history and should not be overlooked. Few guidelines exist regarding the management of chronic thoracic aortic aneurysms specifically; we propose that further research involving patients such as ours is required.

## 4. Patient's Perspective

It was a very surreal feeling when I was diagnosed with a thoracic aortic aneurysm earlier this year, even more so when I found out that the aneurysm originated from a motor vehicle accident back in 1985 and had been present for nearly 30 years. Physically I felt fine, but mentally I suddenly felt vulnerable in terms of the aneurysm rupturing after sneezing or coughing, which I found difficult to get my head around. As a health professional I understood the seriousness of my condition and the complications of the aneurysm rupturing and how lucky I was to have spent 30 years living a care-free life, doing regular and at times very intense exercise and activities. When I was diagnosed, any ache or pain I experienced made me worry that the aneurysm would rupture. After the operation I feel physically no different from how I did before the procedure, but mentally I feel less vulnerable, especially since I have had my follow-up CT which shows no evidence of complication after thoracic aortic stent graft.
